# Immediate and longer term impact of the varicella shortage on children 18 and 24 months of age in a community population

**DOI:** 10.1186/1471-2296-7-51

**Published:** 2006-08-15

**Authors:** Barbara P Yawn, Clayton Schroeder, Peter Wollan, Liliana Rocca, Rick Zimmerman, Barbara Bardenheier

**Affiliations:** 1 Department of Research, Olmsted Medical Center, Rochester, MN, USA; 2 Primary Care, University of Nebraska Medical School, Lincoln, Nebraska, USA; 3 Department of Family Medicine, University of Pittsburg, Pittsburg, PA, USA; 4 Department of Health and Human Services, Centers for Disease Control and Prevention, National Immunization Program, Atlanta, GA, USA

## Abstract

**Background:**

Little is known about the impact of the recent varicella vaccine shortage. To assess the temporal trend in varicella vaccine administration before 18 and 24 months of age in a community cohort of children prior to, during and after the recent varicella vaccine shortage. And to compare the temporal trends in varicella vaccinations to trends of an older, more widely accepted vaccine, the MMR.

**Methods:**

Community population-based birth cohorts were identified who were eligible for the varicella vaccination before, during and after the 2001 to 2002 varicella vaccine shortage. Only children (84% of all) who remained in the community through their second birthday were included. For each child in the cohort, the medical records and immunization registry records from both medical facilities in the county were reviewed to identify the dates and sites for all varicella immunizations given. In addition to varicella immunizations, the dates of all MMR vaccinations were recorded. Additional data abstracted included the child's birth date, gender and dates of any recognized cases of chickenpox up through age 24 months.

**Results:**

Of the 2,512 children in the birth cohorts, 50.8% were boys. In the three cohorts combined, 81.1% of the boys and 79.3% of the girls (p = 0.30) received the varicella vaccine by age 24 months. The pre-shortage community rate of varicella immunization was 79.7% by 24 months of age. During the varicella vaccine shortage, the rate of varicella immunization by 24 months fell to 77.2%. Only 6 additional children received a "catch-up" immunization by 36 months of age. In the post shortage period the community 24-month immunization rate rebounded to a level higher than the pre-shortage rate 84.0%. During the almost three years of observation, the MMR immunization rate by age 24 months was constant (87%).

**Conclusion:**

The varicella shortage was associated with an immediate drop in the 24-month varicella immunizations rate but rebounded quickly to above pre-shortage rates. In this community the only long term impact of the varicella vaccine shortage may be on the small number of children who still had not received catch-up varicella immunizations by 36 months of age.

## Background

Varicella vaccine is a relatively new vaccine that has incomplete but growing acceptance by physicians and parents [1-3]. Currently more physicians recommend the use of varicella vaccine than 5 years ago[4], and more schools and day care sites are requiring receipt of the vaccine for admission[5]. However, the recent extended period of varicella vaccine shortage resulted in temporary modifications of the recommended age for vaccine administration and easing of some requirements for vaccine administration prior to school and day care program entrance[6]. It is not known if this "relaxing" of varicella vaccine requirements will affect clinician's or parent's future acceptance of varicella immunizations or alter the strength of their belief in the benefit of this vaccine[1,2,4,7,8].

The short and long term effects of a vaccine shortage on the rates of a relatively newer vaccine, the varicella vaccine are unknown. Two of the potential short term effects may be an immediate increase in the incidence of the target disease and a failure to provide catch up immunizations leaving a group of children at longer term risk of the target disease. To bolster efforts to provide catch-up immunizations, recommendations for developing recall and reminder programs were distributed through the MMWR and professional medical societies such as the American Academy of Family Physicians[9] and the American Academy of Pediatrics[10]. The shortage of vaccine may also have a longer term impact on decreasing future vaccine acceptance and usage, especially for a vaccine such as varicella that was still in the adoption phase by many parents and clinicians at the time of the shortage[1,2,7,11-17].

Clinicians and parents might feel that the approved delay in the administration of the varicella vaccine suggests it is of lesser value than another vaccine (MMR) that was also in short supply but did not have any modifications for timing of vaccine administration for the first dose or relaxing of requirements for vaccination prior to school entry. The vaccine shortage may therefore affect not only provision of the vaccine during the shortage period but may also decrease or delay administration of the varicella vaccine even after correction of the shortage (longer term effect).

This study addresses the temporal trend in rates of up-to-date varicella immunizations at ages 18 and 24 months in birth cohorts eligible for a varicella and MMR vaccine before, during, and after the varicella shortage period in 2001 and 2002. To place the temporal trends in context, the varicella immunization rates were compared to immunization rates of an older and more widely accepted vaccine, the MMR, that had a similar but less marked shortage problem at about the same time.

## Methods

### Population studied

After approval was received from the Olmsted Medical Center and Mayo Clinic Foundation Institutional Review Boards, all members of three birth cohorts within Olmsted County, Minnesota were selected as subjects for this study. Olmsted County, Minnesota was selected because it has a population-based database that includes all births to Olmsted County residents and all of the health care services, including immunizations used by all members of those birth cohorts. The database includes birth information on 99.5% of all births and health care information on 98% of the residents of the county and covers 98% of the care they receive[18]. The racial diversity of Olmsted County has significantly changed in the past 5 years. Currently the racial and ethnic characteristics of the children born to Olmsted County residents include 65% white non-Hispanic children, 5% Hispanic children, 18% African or Black and 12% Asian (personal communication Division of Health Statistics, Minnesota Department of Health, May 2004). Overall, 23.6% of all children born in Olmsted County to Olmsted County residents are currently receiving Medicaid. Another 15% are covered under the Minnesota subsidized health insurance program, MinnesotaCare.

Because the exact date of the beginning and end of the varicella vaccine shortage period was known for the health care clinics in Olmsted County, Minnesota (December 2001 through August 2002), it was possible to tailor the pre-shortage, intra-shortage and post-shortage birth cohorts to exactly match the shortage period. The birth cohorts correspond to children eligible for the varicella vaccine prior to the shortage (the children who were at 18 to 30 months of age at the beginning of the shortage, December 1, 2001), children primarily eligible for varicella immunization during the shortage (all those who reached 12 months of age during the shortage) and children eligible for varicella immunization after the resolution of the shortage (those who reached 12 months of age after the resolution of the shortage). These three birth cohorts were studied separately to identify up-to-date varicella immunizations by 18 and 24 months of age. The children who were 12 to 18 months during the shortage period were followed out to 36 months of age to see if late catch up immunizations occurred. Children who moved into the community after birth were not included since we had no way of determining the exact age when they entered the community, only when they first accessed health care.

### The setting

Olmsted County is a metropolitan statistical area surrounded by rural agricultural communities and is 90 miles south of the twin cities of Minneapolis and St Paul. The population is relatively isolated from other urban centers and its inhabitants therefore obtain almost all of their primary and tertiary medical care within the metropolitan area of Rochester, Minnesota[18]. The community is unique in that it is relatively isolated and has only two major health care providers both with records that are linked by the diagnostic index of the Rochester Epidemiology Project (REP) that can provide all medical record identifiers for all care used by any child born in Olmsted County.

Children were cared for by 124 family medicine and pediatric physicians and 23 Advance Practice Nurses whose demographics are comparable to those of all non-academic based U.S. primary care physicians (AMA master file, 2002). In Olmsted County the private medical groups were unable to obtain any new doses of varicella vaccine beginning December 1, 2001. The shortage abated in one site by May 6, 2002 but not until August 31, 2002 in the other. MMR vaccine had limited availability only between February 1, 2002 and April 15, 2002. The usual care procedure for both of the two large clinics in Olmsted County, Minnesota (the Mayo Clinic and the Olmsted Medical Center) is to administer the varicella and MMR vaccines simultaneously at 12 to 14 months of age. In the two years preceding the shortage, 80% of varicella immunizations given by 24 months of age were given between 11.5 and 14 months of age and 90% by 15 months of age. Varicella immunization is recommended between 12 and 18 months of age and the MMR between 12 and 15 months of age[5].

### Data collection

All information for this study was collected from the medical records and the immunization registries of the two medical clinics in the county. The information collected included: child's name, mother's maiden name, birth date, gender, date of varicella immunization, date of varicella infection, date of MMR immunization and date of last medical visit in the community. The child's name and mother's name were necessary since children often received care in more than one site, and children's names may change due to marriage or divorce but the mother's maiden name is stable.

### Data analysis

Basic descriptive statistics were used to provide unadjusted immunization rates, calculated separately for each of the 3 cohorts (before, during and after) with binomial confidence intervals. Graphical and tabular summaries are presented.

The primary tool for formal comparison of immunization rates across the three time periods was logistic regression modeling. The response variable was whether or not a child born in Olmsted County in the specified time period of the birth cohort is or is not known to have received varicella vaccination by age 18 (and 24) months. Predictor variables included the time period defining the cohort (before shortage, during shortage or after shortage), and principal site of care. Joint modeling and comparison of MMR and varicella immunization rates was carried out using Generalized Linear Mixed Models (GLMM), adjusting for multiple MMR or varicella immunizations per child. Temporal trends over the birth cohorts were tested using linear and quadratic orthogonal polynomials. A rebound in the vaccination rate corresponds to a significant positive coefficient for the quadratic trend, i.e. a bend departing from a linear time trend. Differences in age at immunization were tested using Wilcoxon rank-sum tests. No formal comparisons between immunization rates at the two large institutions were allowed by either IRB of record.

## Results

A total of 2,512 children were included in the 3 cohorts after removing those who died before age 12 months (n = 25) and those who had primary chickenpox prior to age 18 months (n = 18) (Table 1). The group included slightly more boys (50.8%) than girls (49.2%). Two thirds of the children were born in one of the health system's hospitals and 1/3 in the other.

**Table 1:** Number and gender distribution of children in each phase of the study.

Gender	"Pre-shortage" birth cohort	During shortage birth cohort	Post-shortage birth cohort
Male	742	261	272
Female	726	248	263

The 18 children removed due to primary varicella infection included a total of 17 children (9 boys and 8 girls) who had documented chickenpox prior to age 12 months, and another boy between 12 and 18 months of age.

Between 18 and 24 months only 3 cases of chickenpox were documented in the medical records. Only 1 of these 3 cases of chickenpox occurred during the period of vaccine shortage suggesting that no large number of cases of chickenpox occurred during the vaccine shortage.

The composite rate for varicella vaccination by age 24 months in the three cohorts of children was 82.3%. Of the 1275 boys in the three cohorts, 231 of them received no documented varicella immunization, 24 received a varicella vaccination only after 24 months of age and 81.0% were immunized by 24 months of age. Boys were slightly but not statistically significantly more likely to receive vaccine for varicella than girls (OR 1.06, p = 0.27). Of the 1237 girls, 343 were not immunized, 33 were immunized after 24 months and 80.4% of those eligible were immunized between birth and 24 months of age.

The temporal pattern of varicella vaccination rates at age 18 and 24 months did not vary between the two major health care institutions. Both sites demonstrated the anticipated decline in varicella immunization rates during the shortage, rebounding after the shortage (p = .035). However the decline was greater (6.2%) for the institution that experienced a longer period (9 months, December 2001 through August 2002) of inability to obtain varicella vaccine compared to the institution that experienced only a 4-month period (December 2001 through March 2002) of shortage (0.7% decline).

Not all of the children who failed to receive a varicella immunization during the shortage period had a catch-up immunization by 24 or even by 36 months of age. During the varicella vaccine shortage, the rate of varicella immunization by 18 months fell from 78.1% to 75.2% (p = 0.04). An additional 2% of children in the "shortage cohort" had a catch up immunization between 18 and 24 months of age (varicella immunization rate at 24 months was 77.2%, lower than the pre-shortage 24 month immunization rate of 80.6%, p > 0.05). But of the 127 children who did not receive a catch-up immunization by 24 months, only 6 more received a later "catch-up" immunization by 36 months of age.

**Figure 1:**
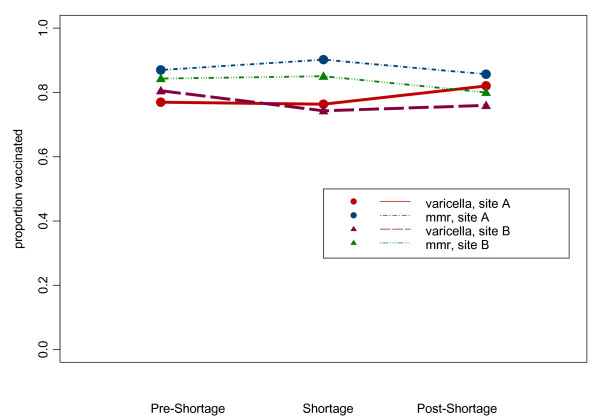
**18-month vaccination rates**. Rate of up to date varicella and MMR immunizations at age 18 months in three cohorts.

**Figure 2:**
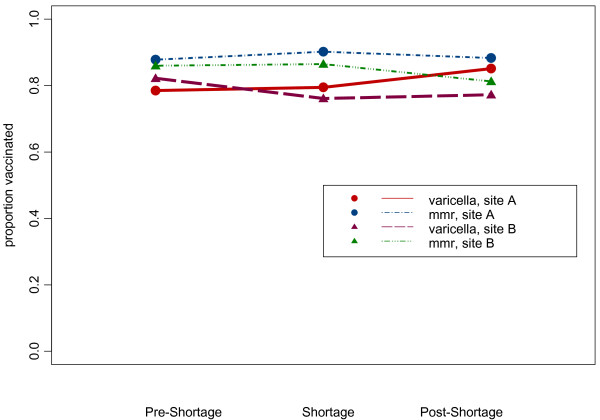
**24-month vaccination rates**. Rate of up to date varicella and MMR immunizations at age 24 months in three cohorts.

In both institutions the varicella immunization up to date rate for children 18 and 24 months rebounded in the post-shortage period. In the institution experiencing the longer shortage, the rate had not yet returned to the pre-shortage rate at the end of the observation period (Figures 1 and 2).

**Figure 3:**
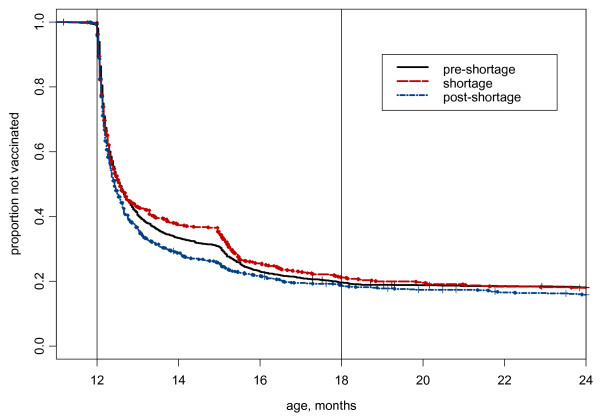
**Survival plots showing time to Varicella Vaccination for 3 birth cohorts**. Comparisons of age at time of varicella vaccination in three birth cohorts.

In an unexpected result, the data indicated that children who received the varicella vaccine in the post-shortage period were being immunized at a slightly younger age than during the pre-shortage period (p < .0001) (Figure 3). For children immunized between 12 and 18 months of age, the mean age at varicella immunization decreased from 395.2 days for the pre-shortage cohort to 386.8 days for the post-shortage cohort. Although the average difference is unlikely to be clinically significant, this group includes 18 children immunized more than 4 weeks before attaining 12 months of age. Of these 18, only two were revaccinated during the required age range.

As expected, the MMR immunization rates by both ages 18 and 24 months were higher than varicella immunization rates at similar ages during all periods studied (p < .0001) (Figures 1 &2). The rate of MMR immunization by age 18 months declined slightly in the post varicella shortage period but was stable by the time children were 24 months of age suggesting that the MMR shortage may have resulted in a slight delay in MMR administration to past 18 months of age in a small group of children.

## Discussion

As anticipated, the varicella vaccine shortage period was associated with an immediate negative but small impact, a decline in the varicella immunization rate appearing not only in the immediate shortage period but persisting up to 36 months of age in the children who were primarily eligible for the varicella vaccine during the shortage period. However, the decline in use of varicella vaccine rebounded following the resolution of the shortage. Therefore, the long-term impact would appear to be limited to any negative longer-term effects for the modest increase in percent of children who were not immunized during the shortage period and did not have catch-up immunizations by 36 months of age compared to birth cohorts before and after the shortage period. This could result in an increase in primary varicella infections or later herpes zoster in this group of children. However, the rate of immunization even in this group did not fall below 70%, which may be sufficient to provide good "herd" immunity[16]. The rate of MMR immunization by 24 months was stable throughout the period of observation. No increase in primary varicella infection rates were seen during the shortage period.

The immediate negative impact on immunizations given to 12 to 18 month old children during the shortage was anticipated nationally and even resulted in modifications of the requirements for varicella immunization prior to day care and school admission for the state of Minnesota. The incomplete catch-up of varicella immunizations in the group that were unable to receive immunizations during the "usual" immunization age period is also not unexpected. The impact on the varicella immunization rate in the cohort following resolution of the shortage is of special interest since these children will require immunization before school entry at age 5 or 6 years but will remain susceptible until that time. Following resolution of the shortage, the varicella immunization rate returned to pre-shortage baseline rates in the entire community but required about 6 months to rebound. It appeared that the rebound returned to the trajectory of overall increase of varicella vaccinations to children 12 to 18 months of age. This increasing trajectory has been seen throughout the U.S. [16]. The other interesting difference is the decline in the average age of the varicella immunization. This appears to parallel the younger age at which the MMR is administered. The implications of this may be that children are making a 12-month "well baby visit" and receiving immunizations. This could be accompanied by a decline in the rate of "well baby" visits at 15 and 18 months since no further immunizations are required. This deserves further study.

Vaccines given too early may not be effective due to interference with maternal antibodies. The ACIP, AAFP, and AAP have published guidelines on the timing of childhood immunizations[19,20]. One study found that 8% of children received at least 1 vaccination dose too early to be considered valid[21]. Our data shows approximately 9% of children vaccinated with varicella vaccine prior to age 12 months.

These study data show that a catch-up varicella immunization process did occur following resolution of the shortage. A group of children (n = 29) received the varicella vaccine between 18 and 24 months, which is later than recommended by the ACIP. Only a few additional children (n = 6) received a late catch-up varicella immunization between 24 and 36 months. This suggests that the recall and reminder systems were not entirely successful in reminding physicians or convincing parents to bring their children back for the immunization. With the current data it is impossible to know which occurred. The benefits of immunization registries in increasing and maintaining higher immunization rates have been well documented in the medical literature [22-26] [Meriwether RA: Proposal for development of a North Carolina immunization registry and plans to improve preschool immunization levels, submitted]. Nothing has been published about the actual functioning of immunization registries in providing "catch-up" immunizations following resolution of a vaccine shortage. These data suggest that studying the functioning of immunization registries in these post-shortage periods could provide important and useful insight into the strengths and weakness of existing immunization registry designs. Do clinicians use the catch-up features of the immunization registries? Are registries adequately designed and equipped to deal with a vaccine shortage or do parents fail to respond to recall or reminder letters once the vaccine shortage is resolved? Solutions are potentially available for each of the proposed problems but the solutions are likely to be very different. This should be further studied.

The stable rate of MMR immunization was anticipated. In the past, the MMR has been more widely accepted by physicians and nurses and parents than the varicella vaccine[1,3]. However, the trajectory of increasing varicella vaccination among all US children (varicella coverage increased from 76% in 201 to 85% in 2003[27]) and the children in this county, shows that by early 2004, the rates of varicella and MMR immunizations are closer than they were in 2001 (3% difference rather than a 6% difference). By 2010, both immunization rates may be close to the Healthy People 2010 goal of 90%[28].

The population in this study is predominantly Caucasian (about 85% white, non-Hispanic). Previous comparison of population-based up-to-date immunization rates in Olmsted County with those from other communities within the US suggest that the findings from Olmsted County immunization studies can be generalized to a large portion of the U. S. population[29]. While caution should be used when generalizing from any single population to that of others, the ability to include an entire community and all primary care physicians and providers rather than the population of a single institution is a major strength of using this community for this study. It is possible that immunizations were obtained at other sites outside the county and therefore we may have under-estimated the vaccination rate during the shortage. However, the entire region of SE Minnesota was experiencing the same shortage and only children taken to other regions of the state or the country would have any greater access to the vaccine. In addition, the local vaccine registries attempt to collect data on "outside" immunizations.

The National Immunization Survey (NIS) also collected data from samples of children in counties throughout the US to assess the impact of the varicella vaccine shortage. However, the NIS had to combine information from many counties since only a few children per county were sampled. This made it difficult to identify associations between the varicella immunization rates and the availability of the vaccine since even adjoining counties often had different levels and durations of varicella vaccine shortage. Therefore, the data from the NIS cannot identify differences in immunization rates based on the severity or duration of the shortage in the county in which the subjects reside. It would be very helpful to have data similar to the data presented here to understand the patterns of impact of the varicella shortage in other areas of the country.

## Conclusion

The varicella vaccine shortage was associated with a small decline in varicella immunization rates during the shortage period. However, the varicella vaccination rate rebounded to pre-shortage rates and appears to continue to be on an upward trend post-shortage.

## Competing interests

The author(s) declare there are no competing interests.

## Authors' contributions

All authors contributed to the design of the study and the securing of funding. BPY oversaw all data collection and PW did all of the data analysis. BPY wrote the original draft of the paper and all authors participated in the critical review of the manuscript and approved the final draft.

## Pre-publication history

The pre-publication history for this paper can be accessed here:



## References

[B1] Ehresmann KR, Mills WA, Loewenson PR, Moore KA (2000). Attitudes and practices regarding varicella vaccination among physicians in Minnesota: implications for public health and provider education. Am J Public Health.

[B2] Niederhauser VP, Baruffi G, Heck R (2001). Parental decision-making for the varicella vaccine. J Pediatr Health Care.

[B3] Schaffer SJ, Bruno S (1999). Varicella Immunization practices and the factors that influence them. Arch Pediatr Adolesc Med.

[B4] Zimmerman RK, Mieczkowski TA, Mainzer HM, Medsger AR, Nowalk MP (2002). Understanding Physician Agreement with Varicella Immunization Guidelines. Preventive Medicine.

[B5] Centers for Disease Control (1999). Prevention of varicella in children. MMWR.

[B6] National Immunization Program http://www.cdc.gov/nip/news/shortages/varicella_02-20-02.htm.

[B7] Yawn BP (1999). Vaccines and Physician Behavior. Arch Pediatr Adolesc Med.

[B8] Rhein L, Fleisher GR, Harper MB (2001). Lack of reduction in hospitalizations and emergency department visits for varicella in the first 2 years post-vaccine licensure. Pediatr Emerg Care.

[B9] American Academy of Family Physicians http://www.aafp.org.

[B10] American Academy of Pediatrics http://www.aap.org.

[B11] Yawn BP, Yawn RA, Lydick E (1997). Community impact of childhood varicella infections. J Pediatr.

[B12] Pathman DE, Konrad TR, Freed GL, Freeman VA, Koch GG (1996). The awareness-to-Fadherence model of the steps to clinical guideline compliance; the case of pediatric vaccine recommendations. Med Care.

[B13] Taylor JA, Newman RD (2000). Parental attitudes toward varicella vaccination. The Puget Sound Pediatric Research Network. Arch Pediatr Adolesc Med.

[B14] DeSerres G, Duval B, Boulianne N (2002). Impact of vaccine cost and information about complications of varicella on parental decision regarding varicella vaccine. Can J Public Health.

[B15] Rothberg M, Bennish ML, Kao JS, Wong JB (2000). Do the benefits of varicella vaccination outweigh the long-term risks? A decision-analytic model for policymakers and pediatricians. Clin Infect Dis.

[B16] Seward JF, Watson BM, Peterson CL, Mascola L, Pelosi JW, Zhang JX (2002). Varicella disease after introduction of varicella vaccine in the United States, 1995–2000. JAMA.

[B17] Vazquez M, LaRussa PS, Gershon AA, Steinberg SPO, Freudigman K, Shapiro ED (2001). The effectiveness of the varicella vaccine in clinical practice. N Engl J Med.

[B18] Melton LJ (1996). History of the Rochester Epidemiology Project. Mayo Clin Proc.

[B19] Centers for Disease Control and Prevention (2002). General recommendations on immunization: recommendations for the Advisory Committee on Immunization Practices and the American Academy of Family Physicians. MMWR.

[B20] Peter G, ed, American Academy of Pediatrics (1997). Active and passive immunization. Red book: report of the Committee on Infectious Diseases.

[B21] Luman ET, McCauley MM, Stokley S, Chu SY, Pickering LK (2002). Timeliness of childhood immunizations. Pediatrics.

[B22] Yawn BP, Edmonson L, Huber L, Poland GA, Jacobson RM, Jacobsen SJ (1998). The Impact of a Simulated Immunization Registry on Perceived Childhood Immunization Status. Am J Man Car.

[B23] Norman LA, Hardin PA, Lester E, Stinton S, Vincent EC (1995). Computer-assisted quality improvement in an ambulatory care setting: A follow-up report. T Comm J Qual Improv.

[B24] Ball TM (1996). Childhood immunizations: Beyond HEDIS. Am J Man Care.

[B25] Crampton RM (1995). The Australian Childhood Immunisation Register (ACIR). Aust Fam Physician.

[B26] Freed GL, Bordley WC, Defriese GH (1993). Childhood immunization programs: An analysis of policy issues. Milbank Q.

[B27] Centers for Disease Control (2004). Immunization registry progress--United States, January-December 2002. MMWR Morb Mortal Wkly Rep.

[B28] U.S. Department of Health and Human Services (2000). Healthy People 2010. With Understanding and Improving Health and Objectives for Improving Health.

[B29] Yawn BP, Edmonson L, Huber L, Poland GA, Jacobson RM, Jacobsen SJ (1998). The Impact of a Simulated Immunization Registry on Perceived Childhood Immunization Status. Am J Managed Care.

